# Utility of Perl’s Prussian Blue Stain in Exfoliated Buccal Cells of Thalassemia and Sickle Cell Anemia Patients and Their Correlation With Serum Ferritin Levels

**DOI:** 10.7759/cureus.47830

**Published:** 2023-10-27

**Authors:** Sahitya Vodithala, Sharadrutha Alampally, Arvind Bhake, Lakshmi Sai Vijay Achalla

**Affiliations:** 1 Department of Pathology, Jawaharlal Nehru Medical College, Datta Meghe Institute of Higher Education and Research, Wardha, IND; 2 Department of Pathology, Prathima Institute of Medical Sciences, Karimnagar, IND; 3 Department of General Surgery, Jawaharlal Nehru Medical College, Datta Meghe Institute of Higher Education and Research, Wardha, IND

**Keywords:** blood transfusion, serum ferritin, iron overload, exfoliative cytology, perl’s prussian blue stain, sickle cell anemia, thalassemia

## Abstract

Introduction

Iron is essential for all living beings. Excess iron, on the other hand, is dangerous because it causes the creation of free radicals. As a result, iron absorption is carefully managed to maintain a balance between absorption and iron loss in the body. Due to the lack of particular excretory channels for iron in humans, iron excess in the tissues is common. It can be caused by a number of factors, including increased iron absorption, as seen in hemochromatosis, or frequent parenteral iron treatment, as seen in thalassemia and sickle cell anemia patients (a transfusional overload).

Aim

The study aims to demonstrate Perl's Prussian blue stain to identify iron overload at a preliminary stage and correlate with serum ferritin levels in patients with thalassemia and sickle cell anemia who frequently receive blood transfusions.

Materials and methods

The present study comprised 62 confirmed cases of thalassemia and sickle cell anemia patients undergoing repeated blood transfusions of a minimum of 15/more, along with 62 clinically healthy individuals between December 2016 and November 2018. The patients with thalassemia and sickle cell anemia were confirmed by hemoglobin electrophoresis (Bio-Rad D-10, Bio-Rad Laboratories, Inc, California, United States). The buccal smears were obtained from these patients along with the controls, and these slides were stained by Perl's Prussian blue stain and were examined under a light microscope.

Results

Sixty-two cases and 62 controls were considered in the current investigation. Forty-seven of the 62 people had thalassemia, and 15 had sickle cell anemia. Thirty-nine out of the 47 patients with thalassemia and six of the 15 individuals with sickle cell anemia had positive results for Perl's Prussian blue stain. All patients had elevated blood ferritin levels, with varying ranges associated with positive results for Perl's Prussian blue stain.

Conclusion

The objective of this study was to demonstrate the utility of oral exfoliative cytology in thalassemia and sickle cell anemia patients who often receive blood transfusions as a screening and diagnostic tool. The exfoliative cytology methods' acceptability and simplicity, along with their correlation with serum ferritin levels and Perl's Prussian blue reaction, make this noninvasive procedure an excellent screening and diagnostic tool for all patients who receive repeated blood transfusions.

## Introduction

Iron is an important nutrient that is required by every human cell. The average adult contains approximately 4.5 g of iron, the majority of which is contained in the hemoglobin molecule and other heme-containing proteins [1]. Under normal physiologic conditions, the concentration of iron in the human body is carefully managed and generally maintained at around 40 mg iron/kg body weight in women and around 50 mg iron/kg body weight in males, with the iron being distributed between functional, transport, and storage components [2].

Iron has the capacity to accept and donate electrons readily, interconverting between ferric (Fe3+) and ferrous (Fe2+). It is a useful component of cytochromes, oxygen-binding molecules (such as hemoglobin and myoglobin), and several enzymes because of this property [3]. These enzymes require the cofactors iron-protoporphyrin (also known as heme) and the iron-sulfur cluster. However, iron can also harm cells by catalyzing the oxidation of hydrogen peroxide and superoxide to free radicals that harm DNA, proteins, and cellular membranes [4]. In a healthy state, there is no detectable concentration of "free iron." Adenosine diphosphate or citrate is used to rapidly chelate any liberated Fe2+ [5]. In developing erythroid precursors and mature red cells, hemoglobin incorporates 80% of the body's iron concentration. About 10-15% of the total is found in muscle fibers (myoglobin) and other tissues (cytochromes and enzymes) [6].

To meet the needs of erythropoiesis, the control of intestinal iron absorption is critical. It is crucial since humans have no physiologic excretory system other than menstruation and enterocyte desquamation (the cycle of enterocytes is around 35 hours). The iron need of the body is sensed by duodenal crypt cells, and the needs of erythropoiesis are programmed by that information as they evolve into absorptive enterocytes. All iron absorption is carried out by enterocytes lining the absorptive villi near the gastro-duodenal junction [7]. The iron balance at the cellular level is achieved by the opposing regulation of ferritin and transferrin receptor production [8].

Patients who depend on blood transfusions and do not obtain appropriate iron chelation therapy frequently have iron overload. The amount of heme iron in each unit of blood is 200 mg, which is more than 100 times the daily average amount of iron routinely absorbed from food. The reticuloendothelial macrophages destroy transfused red blood cells when they senescence and recycle their iron. Following the iron's binding to transferrin, it is released into the bloodstream and disseminated to all bodily tissues [9].

Although hereditary hemochromatosis causes organ damage similar to that found in transfusional iron overload, iron accumulation happens considerably more quickly, and the distribution of iron to reticuloendothelial macrophages is proportionally greater [10]. Transfusional iron overload can usually be recognized from a patient's history if accurate records of transfusions have been kept. After 10-12 transfusions, signs of iron overload are usually visible [9].

Lethargy, weight loss, changes in skin color, a decline in libido, gastrointestinal pain, and joint pain are among the prevalent clinical symptoms of iron overload. Diabetes symptoms arise when the pancreas is affected. Hepatomegaly, skin pigmentation, testicular atrophy, loss of body hair, and arthropathies are the clinical signs [10]. A common conclusive test for determining parenchymal iron overload is liver biopsy. In cases of iron overload involving the reticuloendothelial system, a bone marrow biopsy is advised. However, these invasive procedures might not always be practical [11].

The microscopic examination of cells shed or desquamated from the epithelial surface, usually the mucous membrane, is known as exfoliative cytology. It also includes the examination of cells that have been extracted from bodily fluids like sputum, saliva, etc. It is an easy, quick, bloodless, and painless procedure [12].

In order to enhance the diagnosis and treatment of patients suffering from iron overload resulting from disorders such as thalassemia major, sickle cell anemia, aplastic anemia, myelodysplasia, and hereditary hemochromatosis, among others, a quantitative, noninvasive, secure, reliable, and easily accessible method of measuring body storage iron is needed [13].

This study illustrates iron overload in individuals with sickle cell anemia and thalassemia undergoing blood transfusions using Perl's Prussian blue reaction. A Perl's staining kit consists of potassium ferrocyanide, which reacts with ferritin in the cells to form a blue-colored compound. When viewed under a light microscope, this blue-colored compound appears as blue granules. Thus, the findings of this study can be applied to the early detection of iron overload by a simple, noninvasive method.

## Materials and methods

The study was carried out at Prathima Institute of Medical Sciences, Karimnagar, Telangana, in the Department of Pathology, with permission from the Institutional Ethics Committee named Prathima Institute of Medical Sciences with approval number IEC/PIMS/2016/05. This investigation was carried out both prospectively and retrospectively in the institute during a two-year period from December 2016 to November 2018.

The buccal smears were obtained from patients with thalassemia and sickle cell anemia who were confirmed by hemoglobin electrophoresis (Bio-Rad D-10, Bio-Rad Laboratories, Inc., California, United States) and who required a minimum of 15 or more blood transfusions. The procedure was clearly explained to the patients before performing it in their own language, and the importance of this test was also explained to them. Clinically and hematologically healthy adults without acute or chronic liver injury, cancer, or megaloblastic anemia comprised the control group.

Sample size

A total of 62 cases of thalassemia and sickle cell anemia were taken.

Method of selection

Patients who were already diagnosed with thalassemia and sickle cell anemia and on regular blood transfusions (15/more times) were included in the study.

Inclusion criteria

The inclusion criteria comprised people with thalassemia and sickle cell anemia who require frequent blood transfusions (15 times or more).

Exclusion criteria

The exclusion criteria comprised healthy individuals without a history of malignancy, megaloblastic anemia, or chronic or acute liver disease and people with sickle cell anemia and thalassemia who do not regularly receive blood transfusions.

Method of obtaining buccal smears

The glass slides used to prepare the smears were clean, new, and dry. Patients in the study and control groups were told to gargle with purified water. A wooden spatula soaked with regular saline was used to remove samples from the subject's mouth. Both the study and control groups had their buccal mucosa smears obtained by scraping cells with a gentle motion and minimal pressure. After positioning the scraps in the middle of the glass slides, they were instantly fixed in 90% ethanol for one hour. After that, they were stained with Perl's Prussian blue stain, composed of potassium ferrocyanide and HCl, which produces blue granules when they react with the ferritin in the cells. The positivity of the reaction in our study group was compared with Perl's reaction positivity in iron overload tissues like liver biopsies.

Recording serum ferritin

For both the study group and control group, blood samples from the antecubital vein were taken to estimate the serum ferritin levels using the Abbott i1000SR (Abbott, Illinois, United States) immunoassay.

## Results

In the current investigation, a total of 62 cases of thalassemia and sickle cell anemia, along with 62 controls, were considered. Among the total 62 cases, 47 cases were diagnosed as thalassemia, and 15 cases were of sickle cell anemia. The distribution of the number of cases of thalassemia and sickle cell anemia is shown in percentages in Table 1.

**Table 1:** Distribution of the number of cases (N) of thalassemia and sickle cell anemia in percentages

Serial number	Type of disorder	Number of cases (N)	Percentage of cases
1	Thalassemia	47	75.80%
2	Sickle cell anemia	15	24.19%
3	Total	62	100%

Out of the total 62 cases, 45 cases (72.5%) of thalassemia and sickle cell anemia together showed a positive Perl's Prussian blue reaction, and 17 cases (27.4%) showed a negative Perl's reaction. None of the controls (0%) showed a positive Perl's reaction. The distribution of cases and controls, along with their positive and negative percentage of cases for Perl's Prussian blue reaction, is depicted in Table 2. When the Chi-Square test was applied, the test was considered significant as the p-value was less than 0.0000001 (p<0.05 is considered significant).

**Table 2:** Comparison of Perl's Prussian blue stain in thalassemia and sickle cell anemia cases and controls (p<0.05 is considered significant)

Group	Perl’s Prussian blue reaction					
	Positive		Negative		Chi-square test	p-value
	N	%	N	%	70.63	p<0.0000001
Patients	45	72.58%	17	27.41%		
Controls	00	0%	62	100%		

In our study, exfoliated cells from the buccal mucosa of 39 (82.9%) out of 47 thalassemia patients and 6 (40%) out of 15 sickle cell anemia patients tested positive for Perl's Prussian blue reaction containing blue-colored granules. Eight cases (17.02%) of thalassemia and nine cases (60%) of sickle cell anemia showed a negative reaction to Perl's stain. No controls (0%) showed a positive reaction for Perl's Prussian stain. The percentages of positive and negative cases of iron distribution in exfoliated buccal cells of the cases and controls, along with their percentages, are shown in Table 3.

**Table 3:** Percentages (%) of iron distribution in exfoliated buccal cells of patients with thalassemia, sickle cell anemia patients, and control subjects

Group	Percentages of iron overload in exfoliated buccal cells of cases and controls		Total
	Positive	Negative	
Thalassemia	39 (82.97%)	08 (17.02%)	47 (100%)
Sickle cell anemia	06 (40%)	09 (60%)	15 (100%)
Controls	00 (0%)	62( 100%)	62 (100%)
Total	45 (36.29%)	79 (63.70%)	124 (100%)

The serum ferritin levels of thalassemia cases and controls were calculated. The serum ferritin levels, when calculated, were significantly higher in the study group as compared to the control subjects. Among the cases of thalassemia, the maximum level of serum ferritin observed was 9027 µg/lt, and the minimum level was 554 µg/lt. Among controls, the maximum level of serum ferritin noted was 144 µg/lt, and the minimum level was 35 µg/lt. The mean±SD serum ferritin level in thalassemia patients and control subjects was 2735.46±1877.11 and 89.06±29.54, respectively. The t-test and p-value were obtained. The p-value was less than 0.05, and the test is considered significant. The comparison of serum ferritin levels of thalassemia patients and control subjects, along with their p-values, is shown in Table 4.

**Table 4:** Comparison of serum ferritin levels of thalassemia patients and control subjects (mean±SD); a p-value of <0.05 was considered significant

Serum ferritin levels	Thalassemia patients				Controls				t-test	p-value
	Mean	SD	MIN	MAX	Mean	SD	MIN	MAX	594.471	<0.0000001
	2735.46	1877.11	554	9027	89.06	29.54	35	144		

Similarly, the serum ferritin levels of sickle cell anemia cases and controls were calculated. The serum ferritin levels, when calculated, were significantly higher in the study group as compared to the control subjects. The maximum level of serum ferritin in sickle cell anemia patients observed was 4800 µg/lt, and the minimum level was 530 µg/lt. Among controls, the maximum level of serum ferritin noted was 133 µg/lt, and the minimum level was 38 µg/lt. The mean±SD serum ferritin level in sickle cell anemia patients and control subjects was 1253.06±845.74 and 77.46±28.60, respectively. The t-test and p-value were obtained. The p-value was less than 0.05, and the test is considered significant. The comparison of serum ferritin levels of sickle cell anemia patients and controls, along with their p-values, is shown in Table 5.

**Table 5:** Comparison of serum ferritin levels of sickle cell anemia patients and control subjects (mean±SD); a p-value of <0.05 was considered significant

Serum ferritin levels	Sickle cell anemia patients				Controls				t-test	p-value
	Mean	SD	MIN	MAX	Mean	SD	MIN	MAX	5.39047	<0.0000001
	1253.06	845.74	530	4800	77.46	28.60	38	133		

**Figure 1:**
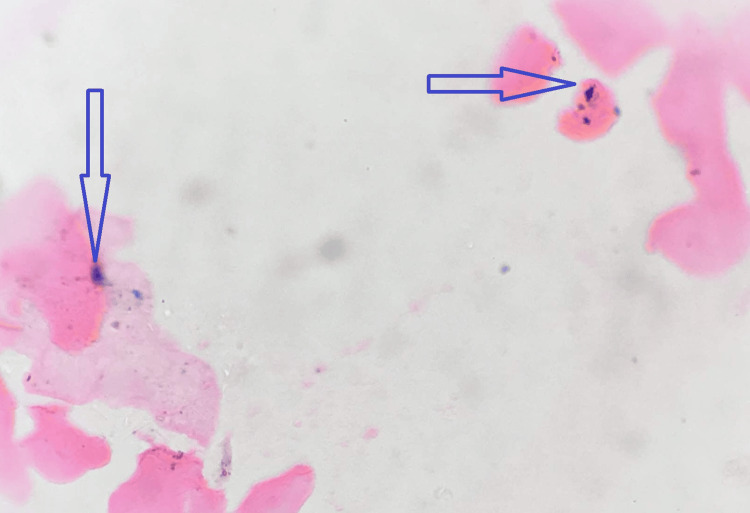
Pictomicrograph showing fine dispersed intracellular blue-colored granules in oral exfoliated cells of thalassemia patients (Perl's Prussian blue stain, x100)

Upon staining the buccal smears with Perl's Prussian blue stain, these exfoliated cells showed the presence of blue-colored, finely dispersed to mild clumps of intracellular granules under x100 and x400 magnification, which are considered a positive reaction. Figure 1 shows the pictomicrograph showing Perl's Prussian blue stain positivity in exfoliated buccal mucosal cells of patients with thalassemia.

**Figure 2:**
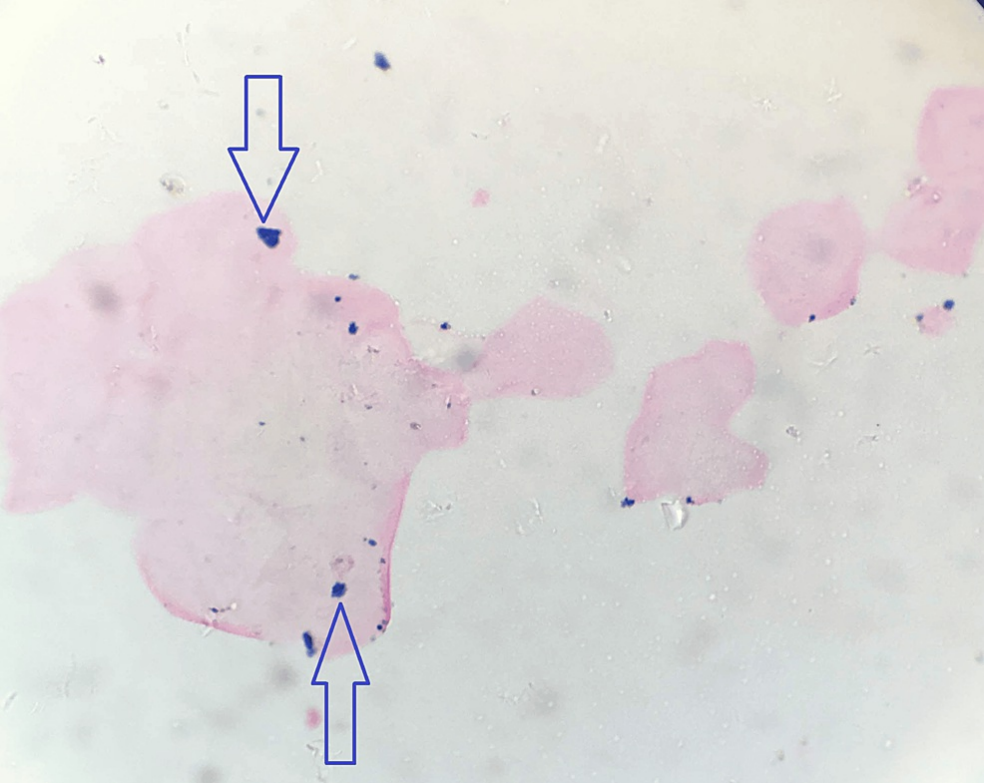
Pictomicrograph showing blue-colored fine intracellular granules in oral exfoliated cells of sickle cell anemia patients (Perl's Prussian blue stain, x100)

Similarly, on the staining of the buccal smears of sickle cell anemia patients with Perl's Prussian blue stain, these exfoliated cells showed the presence of fine dispersed to mild clumps of intracellular granules under x100 and x400 magnification, which are considered positive reactions. Figure 2 shows the pictomicrograph showing Perl's Prussian blue stain positivity in oral exfoliated buccal cells of sickle cell anemia patients.

## Discussion

In the current investigation, an effort was made to correlate the levels of serum ferritin in patients with thalassemia and sickle cell anemia to the presence of iron in the exfoliated oral epithelial cells as the ferric iron is liberated from unreactive loose combinations with proteins such as hemosiderin. This technique was applied to exfoliated buccal mucosal cells, considering the fact that exfoliated buccal cells possibly represent the changes in the underlying parent tissue. It was found that the iron excess in the oral mucosal cells of the thalassemia patients increased along with the serum ferritin levels, which was statistically significant (p<0.001). This suggests that the presence of positive staining in exfoliated buccal cells may be used to detect changes in serum ferritin levels and, consequently, iron excess in the bodily tissues.

Patients with sickle cell anemia and thalassemia accumulate iron based on the number of blood transfusions they receive. About 250 ml of RBCs make up one unit of blood. Since 1 ml of RBC contains 1 mg of iron, four units of blood will roughly contain 1 g of iron. Clinical toxicities manifest when the body's iron content reaches 400 to 1000 mg/kg of body weight. The signs of an iron overload can usually be seen after 10-12 transfusions [7]. Because oral exfoliated cells may reflect changes in the parent tissue, they can be examined for pathologic abnormalities associated with the parent tissue. Using Perl's Prussian blue reaction, this technique is used to illustrate the iron in the oral exfoliated cells of persons with iron excess [8].

The gold-standard, conclusive test for determining parenchymal iron overload is a liver biopsy. In cases of reticuloendothelial system-related iron overload, a bone marrow biopsy is advised. However, these procedures are invasive, and they might not always be practical [14]. Oral exfoliative cytology is a rapid, painless, and safe way to measure the body's iron levels using Perl's Prussian blue stain [9-11].

The buccal smears of 45 patients in the study group tested positive for Perl's reaction. When the "p" value was less than 0.05, it was considered statistically significant. Gururaj et al. [11] investigated the presence of iron in exfoliated oral mucosa in 10 thalassemia patients receiving frequent blood transfusions in 2003. The blue granules were seen in the oral exfoliated cells of buccal smears of all patients, i.e., in 100% of the patients.

Nandprasad et al. [15] exhibited oral exfoliative cytology in their investigation of 100 patients with β-thalassemia who received repeated blood transfusions, a minimum of at least 15 transfusions. Of the 100 individuals with significant β-thalassemia, 65 had exfoliated cells from their buccal mucosa that tested positive for Perl's Prussian blue reaction. Using the Chi-square test to get the p-value, the results clearly showed that the p-value was <0.005 and considered significant.

In 2013, Bhat et al. [16] determined the presence of iron in exfoliated buccal cells of β-thalassemia major patients. The buccal smears of a total of 60 randomly chosen β-thalassemia major patients (22 females and 38 males) were obtained. About 71.7% of thalassemic patients showed Perl's positivity, which correlated with serum ferritin levels.

In a 2013 study, Chittamsetty et al. [17] investigated a noninvasive method that shows the iron in the buccal mucosa of patients with sickle cell anemia and thalassemia who received frequent blood transfusions. Twenty patients with sickle cell anemia and 40 patients with β-thalassemia major receiving repeated blood transfusions of at least 15 or more were included in the study. After staining the buccal smears with Perl's stain, it was observed that 29 (72.5%) of the 40 (100%) thalassemia patients tested positive for iron overload, and 11 (27.5%) tested negative. Thirteen (65%) of the 20 (100%) sickle cell anemia patients tested negative for iron using Perl's stain, while seven (35%) showed positive results. None of the 60 controls exhibited positive. It was observed that the p-value was less than 0.05 after using the Chi-square test. Therefore, the distribution of iron excess was statistically significant in patients with sickle cell anemia and thalassemia.

In the present study, of the 47 (100%) thalassemia patients, 39 (82.97%) were iron positive and eight (17.02%) were iron negative, and of the 15 (100%) sickle cell anemia patients, six (40%) were positive and nine (60%) were negative for Perl's stain. None of the 62 controls displayed positive staining for Perl's Prussian blue. It was observed that the p-value was less than 0.00000001 when the Chi-square test was applied, and it was considered statistically significant (p<0.05).

Only six (40%) of the 15 sickle cell anemia patients tested positive for iron in Perl's Prussian blue reaction in the current investigation. This could be due to the patients receiving fewer blood transfusions in comparison, which resulted in less iron overload in the oral mucosal cells, as well as the small sample size and the wide range of variations in serum ferritin levels (530-4800 gm/lt).

The comparison of Perl's Prussian blue stain positivity of various studies with the present study is shown in Table 6.

**Table 6:** Comparison of Perl's Prussian blue stain percentage positivity of various studies with the present study

Sr. no	Study	Year	Positive cases	% positivity
1	Gururaj et al. [11]	2003	10/10	100%
2	Nandprasad et al. [15]	2010	65/100	65%
3	Bhat et al. [16]	2013	43/60	71.7%
4	Chittamsetty et al. [17]	2013	29/40	72.5%
5	Sonamgupta et al. [18]	2014	37/60	61.6%
6	Rathore et al. [19]	2016	29/35	82.9%
7	Leekha et al. [20]	2016	35/40	87.5%
8	Gajaria et al. [21]	2017	49/50	98%
9	Present study	2018	45/62	72.58%

Furthermore, none of the patients in the control groups of the studies conducted by Gururaj et al. [11], Nandaprasad et al. [15], and the current study exhibited a positive Perl's reaction.

Because of an excess of alpha globin chains and an increased rate of red cell disintegration, β-thalassemia causes inefficient erythropoiesis, which results in severe anemia. They consequently need frequent blood transfusions. The goals of transfusion are the correction of anemia, the reduction of iron absorption via the gastrointestinal tract, and the suppression of erythropoiesis [14].

There are a few approaches to investigating the iron status of individuals with transfusional iron overload, but none of them yields good, satisfying results. To characterize the quantity of iron and its distribution to various organs, two or more iron indices will typically be needed. Broadly speaking, two categories of techniques can be distinguished: direct techniques, which rely on identifying iron in the tissue through biopsy or noninvasive procedures, and indirect techniques, such as measuring the concentration of serum ferritin and iron saturation of serum transferrin [22].

The combination of regular blood transfusions and chelation therapy has extended the life expectation of thalassemia patients into their fourth and fifth decades. On the other side, numerous blood transfusions have resulted in iron excess and a variety of problems, the most prevalent of which is growth failure. Growth problems are also a common clinical symptom in untreated thalassemia patients [23].

Transfusions enhance blood flow in sickle cell disease patients by lowering the percentage of red blood cells that can form sickle hemoglobin polymer. This reduces hemolysis and endothelial damage caused by elevated levels of red blood cells carrying sickle polymer [23].

Transfusion is well defined as both prophylactic (stroke) and therapy (acute chest syndrome and stroke) for significant sickle cell disease sequelae. Iron overload is the most common and unavoidable side effect of sickle cell disease transfusions [23]. Although transfusion is a commonly used therapy in sickle cell disease, its most well-established uses have been in preoperative prophylaxis, acute chest syndrome treatment and prophylaxis, and stroke treatment [24-26].

Although transfusion can help with medical problems, overabundance of iron is an unavoidable and dreaded adverse effect of persistent transfusion therapy. Patients with sickle cell disease who received chronic transfusions for iron excess exhibited a significantly higher death rate than the patients without iron overload [27]. Despite a similar transfusion history, patients with sickle cell disease are considerably less likely to be tested for end-organ damage than patients with thalassemia [28].

## Conclusions

The purpose of this study was to establish oral exfoliative cytology as an ideal screening and diagnostic method in patients with thalassemia and sickle cell anemia who often receive blood transfusions.

This noninvasive technique, by considering the acceptability and ease of use of exfoliative cytology techniques along with the correlation between serum ferritin levels and Perl's Prussian blue reaction, can be established as the best screening and diagnostic method for all those who receive frequent blood transfusions. They can also predict potential problems caused by an excess of iron, which will aid in selecting the most appropriate treatment.

This study is a qualitative study that evaluated only the presence or absence of an iron overload in the oral mucosal cells of patients with thalassemia and sickle cell anemia, but the amount of iron overload cannot be measured quantitatively. To demonstrate that this noninvasive method is the best screening and diagnostic tool for all patients needing frequent blood transfusions, larger sample volumes and a quantitative evaluation of the iron overload may be necessary.
